# Therapeutic hypothermia in an out-of-hospital arrest population: are we selecting appropriately?

**DOI:** 10.1186/cc10887

**Published:** 2012-03-20

**Authors:** A Short, M Brett, L Donaldson

**Affiliations:** 1 Glasgow Royal Infirmary, Glasgow, UK

## Introduction

We question how appropriately we select patients to undergo therapeutic hypothermia following out-of-hospital cardiac arrest.

## Methods

The population was identified through searching Wardwatcher between August 2006 and February 2011. Inclusion criteria were all patients with an ICU admission of out-of-hospital cardiac arrest. Exclusion criteria were: no CPR within the preceding 24 hours; admission from theatre; insufficient data. Data were gathered from Wardwatcher, Careview and patients' case notes for age, arrest rhythm, downtime (DT) - time from arrest to return of spontaneous circulation, time to initiation of CPR, temperatures at various time points, cause of arrest and outcome. Statistical analysis was performed with Fisher's exact test, significance level of *P *< 0.05. Permission for use of patient notes was granted from the consultant group of the ICU audited.

## Results

Seventy patients had a hospital admission of post-cardiac arrest. Five failed the inclusion criteria and six fulfilled exclusion criteria. A total of 36 (51%) were cooled (Table 1). Twelve (33%) of the cooled population survived to hospital discharge (D/C), one (8%) cooled within 4 hours, three (25%) cooled for over 12 hours. Ten (28%) patients were cooled despite not having a cardiac cause. One (4%) of the 23 noncooled patients survived to hospital discharge, four (17%) had a cardiac cause. The median age of cooled population was 66 years (quartile range 53.5 to 74 years) and 44 years (quartile range 41 to 52 years) of the noncooled.

**Table 1:** Population cooled post cardiac arrest

	D/C (*n *= 12)	Died (*n *= 26)	
VF arrest	10 (83%)	18 (75%)	*P *= 0.69
DT >30 minutes	1 (8%)	14 (58%)	*P *= 0.005
First CPR <5 minutes	11 (92%)	17 (71%)	*P *= 0.22
Cardiac aetiology	10 (83%)	16 (67%)	*P *= 0.44

## Conclusion

Survival is improved in patients cooled post-out-of-hospital cardiac arrest [1,2]. Downtime is statistically significant in the survival of cooled patients. Achieving optimal timing of cooling was no better in surviving versus dying populations. Cooling post-out-of-hospital cardiac arrest is expensive and time-consuming; selection criteria need to be evaluated to concentrate this resource on patients where there is a higher prospect of a positive outcome [2].

## References

[B1] Holzer M Crit Care Med 2005 33 414 418 10.1097/01.CCM.0000153410.87750.53 15699847

[B2] Hay A Anaesthesia 2008 63 15 19 18086065 10.1111/j.1365-2044.2007.05262.x

